# Role of Exosomes in Immune Microenvironment of Hepatocellular Carcinoma

**DOI:** 10.1155/2022/2521025

**Published:** 2022-01-28

**Authors:** Xiaojing Chen, Hao Chi, Xiaozhao Zhao, Rui Pan, Ying Wei, Yunwei Han

**Affiliations:** ^1^Department of Oncology, The Affiliated Hospital of Southwest Medical University, Luzhou, 646000 Sichuan, China; ^2^Clinical Medical College, Southwest Medical University, Luzhou, 646000 Sichuan, China

## Abstract

Hepatocellular carcinoma (HCC) is the most common primary liver cancer. Since most patients with HCC are diagnosed at the intermediate or advanced stage and because HCC has a high incidence of metastasis and recurrence, it is one of the leading causes of cancer death. Exosomes are a subtype of extracellular vesicles and are typically 30–150 nm in diameter. Originating from endosomes, they can be secreted by almost all living cells. They are widely present in various body fluids and serve as an important medium for the interactions between cells. A series of studies have revealed that exosomes-mediated intercellular transfer of proteins, nucleic acids, and metabolites plays a crucial role in the initiation and progression of HCC, hypoxia and angiogenesis, chemotherapy sensitivity, and cell death mode and regulates the immune microenvironment. In this paper, we reviewed the recent researches on the multiple roles of tumor-associated exosomes in the progression of HCC. We laid particular focus on those researches that reveal how exosomes regulate the tumor immune microenvironment (TIME) and how exosomal cargos affect the progression of HCC. Besides, we emphasize some prospective directions to achieve a more accurate and complete analysis of the HCC immune microenvironment.

## 1. Background

Hepatocellular carcinoma (HCC) is the sixth most common cancer in the world and the third most fatal cancer [1]. Despite great breakthroughs in HCC treatments, such as surgical resection, radiotherapy, and immunotherapy, the prognosis remains poor. This is mainly because most HCC patients are diagnosed at the intermediate and advanced stages and lack specific biomarkers, therefore missing out on the optimal treatment window. Recently, attention has focused on immunotherapy for it greatly activates autoimmunity. Unfortunately, its therapeutic performance in clinical trials has been unsatisfactory. This is associated with the formation of the HCC immunosuppressive microenvironment which seriously hinders the antitumor effect of immune checkpoint inhibitors, attenuating the efficacy of immunotherapy [2]. At present, the mechanism for the formation and regulation of the immunosuppressive microenvironment remains unclear.

Exosomes are the smallest subtype of extracellular vesicles (EVs), ranging in diameter from 30 to 150 nm with a density of 1.13 to 1.19 g/ml [3]. Regardless of the type or the physiological or pathological state of the originating cell, exosomes share common features as enriched with nucleic acids, lipids, and proteins [4]. In addition, the exosomes released from the same cell line can be highly homogeneous [5]. But the specific exosomal composition depends to some extent on the cell type and is also affected by different cell conditions or treatments. Compared with normal cells, tumor cells secrete more exosomes, and the number of exosomes secreted by different tumor cells varies dramatically [6]. And thanks to the lipid bilayer structure of exosomes, cargos contained in them are not easily degraded so as to convey cargos stably [7–9]. With the properties listed above, exosomal cargos have the potential to be used as biomarkers, therapeutic targets, or immunomodulators.

A number of studies have proved that exosomes are important mediators for the initiation, progression, angiogenesis, and metastasis of tumors [10]. Exosomes act as a signal-transmitting medium between cells to mediate intercellular communication and change the functional status of recipient cells [11]. In particular, the exosomal nucleic acids are usually accurately quantified after the exosomes are isolated, and their profiles can accurately reflect a variety of physiological conditions and diseases [12,13]. HCC-derived exosomes participate in changing tumor progression by regulating the tumor microenvironment and tumor immune status [14,15]. Therefore, the current research focuses on the interaction between exosomes and immune cells in HCC.

## 2. Biogenesis and Acquisition of Exosomes

### 2.1. Biogenesis and Accepting of Exosomes

Exosomes were first described in 1981 as ATPase-containing vesicles exfoliated from cells and were found in various normal cells and tumor cells [16]. The biogenesis of exosomes begins in the endosome. First, endocytic vesicles arise when the cellular membranes invaginate, and they fuse together to create early endosomes. After the early endosomes mature, they become late endosomes, namely, MVB. In the process, the early endosome membrane buds inwards and encapsulates the intracellular material, producing intraluminal vesicle (ILV) in the lumen of the organelle [17]. The biogenesis of exosomes mainly involves endosomal sorting complexes required for transport protein (ESCRT). ESCRT includes ESCRT-0, ESCRT-I, ESCRT-II, ESCRT-III, and related AAA-ATPase Vps4 complex [4]. Through its ubiquitin-interacting domain, ESCRT-0 drives the concentration and transfer of exosomal cargos and further recruits other ESCRT proteins, such as ESCRT-I, ESCRT-II, and ESCRT-III, to the endosomal membrane [18,19]. After the ESCRT complex is assembled, ESCRT-I and ESCRT-2 are responsible for the budding of endosomal membranes. Then ESCRT-III catalyzes the scission of necks of ILVs by forming polymer filament and finishing the inward budding [20,21].

The sorting of exosomal cargos and the release of exosomes also require the involvement of apoptosis-linked gene-2 (ALG-2), interacting protein *X* (Alix), tetraspanins (CD9, CD63, and CD81), and tumor susceptibility gene 101 protein (TSG101). Finally, the secretion of exosomes entails the participation of SNARE protein complexes and synaptic binding proteins. The MVB moves toward the cell surface, fuses with the cellular membrane, and afterwards releases the ILVs into the extracellular milieu as exosomes, which are then known as exosomes and enter into various body fluid environments [22,23]. In addition, studies have revealed the ESCRT-independent exosomal pathway, which depends on ceramide and tetrahydrocannabinol [24,25]. More notably, it is discovered that RAB31 has two roles in exosomes biogenesis: it drives the production of ILVs and protects MVB from degradation. The active RAB31, phosphorylated by multiple RTKs, interacts with flotillin proteins contained in lipid raft microdomains to induce the budding of the MVB membrane and then the formation of ILVs. Meanwhile, RAB31 attracts TBC1D2B to deactivate RAB7, inhibiting MVBs from merging with lysosomes and allowing ILVs to be secreted as exosomes [26].

**Figure 1:**
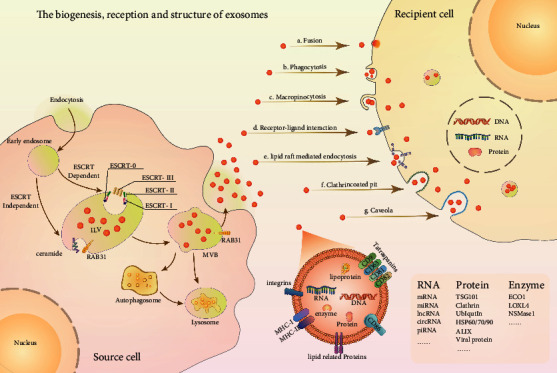
Schematic illustration of the biogenesis, secretion, absorption, and composition of exosomes. Exosome biogenesis initiates with the process of inward budding of the cell membrane, which is called endocytosis, followed by the formation of endosomes. Then the ILVs were formed by the invagination of the endocytic membrane into the organelle lumens; thus the endosomes turn into MVBs. The MVBs fuse with the plasma membrane through ESCRT-dependent or ESCRT-independent pathways to release exosomes, otherwise, fusing with lysosomes for degradation or ending up to autophagosomes. Subsequently, the released exosomes can be uptaken by recipient cells via fusion, phagocytosis, micropinocytosis, receptor-ligand interaction, lipid raft mediated endocytosis, and clathrin or caveola coated pit. Exosome contains an assortment of bioactive cargos, including RNA, protein, and enzyme.

The released exosomes travel through various body fluids and incorporate by recipient cells by several mechanisms, including direct membrane fusion, phagocytosis, micropinocytosis, receptor-ligand interaction, lipid raft mediated endocytosis, and clathrin or caveola coated pit [4] (Figure 1).

### 2.2. Acquisition of Exosomes from HCC Patients

Almost all cells can secrete exosomes [27] and exosomes are present in various body fluids. The exosomes in the blood can be divided into two major types: serum-derived exosomes and plasma-derived exosomes. Serum exosomal miR-320d [28], LUCAT1 [29], circAKT3 [30], circPTGR1 [31], etc. can be used as markers for the diagnosis and prognosis of HCC. Similarly, plasma exosomal miR-96 [32], lncRNA SENP3-EIF4A [33], circUHRF1 [34], etc. can be used for the diagnosis of HCC as well. But it can be seen that serum exosomes and plasma exosomes as diagnostic molecular markers have certain differences in their composition. The main reason is that, in the process of obtaining serum, platelets in the blood coagulate and then secrete a large number of exosomes [35], which may cause interference in the test results. As a result of this potential interference, it is recommended to choose plasma samples unless the platelet-related disease is the object of study. It is worth noting that the expression of the same molecule in serum exosomes and plasma exosomes may have different significances. Serum exosomal miR-21-5p is correlated with advanced stage and it is an independent prognostic factor for disease-free survival in patients with early HCC [36]. Meanwhile plasma exosomal miR-21-5p can be used as a potential biomarker for the diagnosis of HCC [37].

**Figure 2:**
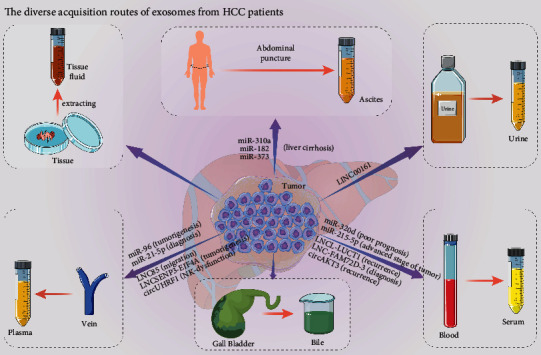
The diverse acquisition routes of exosomes from HCC patients. Exosomes participate in HCC progression and can be extracted from different sources. Exosomal cargos such as small RNA, long noncoding RNA, and circ RNA regulate the malignant phenotypes and can be used as markers for the diagnosis, prognosis, recurrence, migration, and invasion ability of HCC and tumor angiogenesis.

Although current studies of exosomes focus on blood-derived exosomes, we should not ignore the potential of exosomes from other body fluids (Figure 2). Actually, exosomes can be obtained from the urine and ascites of HCC patients. Exosomes from the urine of HCC patients were successfully extracted and it was found that LINC00161 was expressed in urine [38]. Furthermore, it has been found that the expression levels of ascitic fluid-derived exosomal miR-182, miR-301a, and miR-373 were significantly upregulated in liver cancer [39]. As bile is a fluid directly secreted by liver cells, bile-derived exosomes might be more organ-specific and sensitive. Meanwhile, bile exosomal miR-483-5p and miR-126-3p were found to have diagnostic values for malignant biliary obstruction [40]. At present, there is little research related to bile-derived exosomes in HCC patients.

To date, tissue-derived exosomes show unique advantages. By using electron microscopy, researchers found that there are extracellular vesicles of different sizes in the extracellular space of prostate cancer tissues and melanoma tissues and thereby confirmed the existence of exosomes in the interstitial space [41,42]. These extracellular vesicles can reveal the current physiological and pathological conditions of the tissue and participate in the generation of tissue microenvironment [4]. Previously, exosomes from brain tissues digested by collagenase type 3 have been successfully extracted [43]. And then the protocol of enzymatic treatment of dissociated issues followed by differential ultracentrifugation and density separation was proposed. Through this approach, up to six extracellular vesicles subpopulations were isolated, including exosomes [44]. These methods showed that we can extract exosomes from tissues. Whereas exosomes in the blood are derived from multitissue cells, the exosomes secreted by the cells exist first and foremost in interstitial space, which means that tissue-derived exosomes can better reveal the tumor microenvironment. However, there are few studies on exosomes derived from HCC tissues, which leave room for further exploration.

## 3. Exosomal Cargos as Biomarkers or Regulators in HCC

### 3.1. Exosomal DNA

Thanks to the lipid bilayer membrane, which provides exosomes enough space to package relatively large molecular weight DNA and can serve as a highly sensitive and specific biomarker for tumors [45, 46]. Similarly, cell-free DNA (cfDNA) is also considered as a potential diagnostic biomarker. But the detection rate of exosomal DNA in early HCC patients was higher than that of cfDNA [47], because early liver cancer cells derived exosomal DNA can be secreted into body fluids, while cfDNA is derived from damaged, apoptotic, or necrotic cancer cells and its level will not increase significantly until HCC progresses into advanced stage [48]. In addition, pancreatic ductal adenocarcinoma patients revealed that the KRAS mutation rate in exosomal DNA is significantly higher than that in cfDNA [49]. Thus, exosomal DNA may hold more potential for early HCC diagnosis with unique features.

### 3.2. Exosomal mRNA

Efforts are being made to explore the application of exosomal mRNA. The expression level of exosomal hnRNPH1 mRNA in HCC patients increases significantly and is positively correlated with portal vein tumor thrombosis, Child-Pugh score, TNM staging, and lymph node metastasis [50]. Similarly, the serum exosomal RAB11 A mRNA shows high specificity (73.3%) and sensitivity (75%) in distinguishing healthy people, chronic hepatitis C patients, and HCC patients [51]. Apparently, exosomal mRNA can be used as an independent HCC diagnostic marker.

### 3.3. Exosomal miRNA

A large number of studies have analyzed the genetic materials present in exosomes. Among them, the role of Exo-miRNA in HCC is by far the most widely described. Next, we introduce several in-depth studies of hypoxia, angiogenesis, epithelial-to-mesenchymal transition (EMT), chemotherapy sensitivity, etc. and discuss the various roles that exosomal miRNAs can play under different conditions.

#### 3.3.1. Exosomal miRNA Is Involved in Regulating the Hypoxia

**Figure 3:**
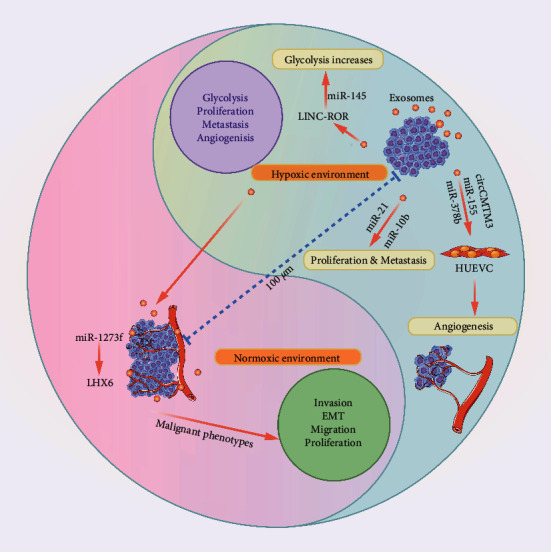
Exosomal miRNA is involved in regulating the hypoxic. Hypoxia usually occurs in solid tumors about 100 *μ*m from the functional vessels. Under hypoxia conditions, tumor cells activate multiple signaling pathways to enhance the release of specific cargos in exosomes and induce angiogenesis, glycolysis, self-proliferation, and metastasis, thereby adjusting their adaptability to hypoxia environment. Additionally, they promote the malignant phenotype of normoxic tumor cells and lead to disease progression.

The diffusion limitation of oxygen and the anatomical or physiological abnormalities of tumor vasculature can cause varying degrees of hypoxia in solid tumors. Tumor cells around normal functional blood vessels are viable and proliferative, while solid tumors about 100 µm away from functional blood vessels usually exhibit hypoxia [52]. And hypoxia is the dynamic and heterogeneous characteristic of most solid tumors [53], while hypoxic tumor cells integrate existing blood vessels nearby or form new blood vessels by inducing tumor angiogenesis factors, thereby establishing a new tumor microenvironment [54]. Under hypoxic conditions, tumor cells secrete a fairly great number of exosomes. Whereas hypoxia in normal cells leads to cell death, hypoxia in tumor cells can induce specific exosomes release to adapt to the unfavorable microenvironment (Figure 3). For example, exosomal miR-155, miR-378b, and circCMTM3 derived from HCC cells promote angiogenesis in human umbilical vein endothelial cells under hypoxic conditions [55–57]. Interestingly, HCC-derived exosomal miR-3682-3p as a negative regulator of angiopoietin-1 inhibited endothelial cells resulting in angiogenesis impairment; consequently, tumor cells downregulated its expression [58].

Energy metabolism reprograms through exosomes and impels tumor cells to achieve malignant proliferation with hypoxia. Under hypoxic conditions, HCC cells promote the secretion of exosomal linc-RoR, which can activate the HIF-1*α*, thereby enhancing the glycolysis to resist the hypoxic environment [59]. Typically, glycolysis is a prominent feature of tumors under hypoxic conditions, while another major feature is the low PH caused by high levels of lactic acid [60]. Then the acidic microenvironment triggers the expression of miR-21 and miR-10b in the exosomes of HCC cells and promotes proliferation and metastasis [36]. In addition, exosomal cargos can also induce normoxic cells to establish malignant phenotypes. Hypoxia cells are induced to produce more exosomes and then promote the expression of miR-1273f in normoxic HCC cells targeting LHX6 for downregulation, thereby enhancing the EMT and metastasis of normoxic HCC cells [61].

#### 3.3.2. Exosomal miRNA Regulates the EMT Procession

EMT is the process of epithelial cells transitioning into mesenchymal cells with migration and invasion properties, which are closely related to tumor metastasis [62]. Exosomes containing miRNA can induce EMT in HCC. Exosomal miR-24-3p produced after SMMC-7721 cells treated with transforming growth factor *β* (TGF-*β*) undergo EMT can promote the migration and invasion of not treated SMC-7721 cells [63]. Besides, exosomal miRNA-451a derived from HUCMSCs can inhibit ADAM10 to decrease EMT in HCC [64]. At the same time, the exosomes derived from HCC cells can also mediate EMT through TGF-*β*/Smad and MAPK/ERK signaling pathways [65, 66]. Mesenchymal-epithelial transition (MET) is EMT in reverse and a large number of studies have shown that MET also plays an important role in the metastasis of breast cancer, pancreatic cancer, and other tumors [67]. Whether these roles are present in HCC, however, requires further research.

#### 3.3.3. Exosomal miRNA Regulates the Chemotherapy Sensitivity

To date, HCC shows high resistance to commonly used chemotherapy drugs such as fluorouracil, doxorubicin, and cisplatin [68]. Recent studies have shown that exosomal miRNAs are related to cancer chemosensitivity [69, 70]. Exosomal miR-451a can increase the chemosensitivity of HCC to paclitaxel by targeting ADAM10 [64]. Similarly, miR-199a can modify the exosomes from adipose-derived mesenchymal stem cells and thereafter increase the sensitivity of HCC cells to doxorubicin via the mTOR pathway [71]. Exosomes from miR-122-modified adipose-derived mesenchymal stem cells (AMSCs) can inhibit the expression of ADAM10, IGF1R, and CCNG1, thereby increasing the chemosensitivity of HCC cells to sorafenib [72]. Analogously, exosomal miR-744 inhibits the proliferation of HCC cells by targeting PAX2 and increases the sensitivity to sorafenib [73]. Furthermore, exosomes derived from human embryonic kidney epithelial cells function as nanoparticles to transfer miR-199a-3p, thereby reversing the chemoresistance of HCC cells to cisplatin [74].

In addition to improving the sensitivity of HCC to chemotherapy drugs, exosomal miRNAs can also increase drug resistance. For example, adipose-derived exosomal miR-23a and miR-23b confer HCC cells with chemoresistance to fluorouracil [75]. The tumor suppressor gene PTEN is often associated with acquired resistance to drugs [76, 77]. The exosomal miR-32-5p derived from multidrug-resistant HCC cells inhibits PTEN and activates the PI3K/Akt pathway, thereby inducing multidrug resistance further to transform sensitive cells into resistant cells [78].

In short, exosomes can mediate the chemotherapy sensitivity or resistance of tumor cells through miRNAs. Meanwhile, the above evidence also indicates that many exosomes derived from normal cells are vital players in regulatory role in the chemotherapeutic sensitivity of HCC. As a result, the exosomes of normal cells, functioning as delivery vehicles to improve the sensitivity of HCC chemotherapy which could be a new strategy to inhibit HCC, are worthy of our attention.

#### 3.3.4. Exosomal miRNA Regulates the Cell Death Methods

Some researchers divide the cell death methods roughly into two types: accidental cell death (ACD) and regulated cell death (RCD). According to the morphological, biochemical, and genetic characteristics of various cell death modes, apoptosis, autophagy, pyrolysis, and ferroptosis can be classified as RCD and necrosis as ACD [79]. The extensive role of exosomal miRNA has been shown in a variety of cell death mechanisms, which consist of apoptosis, autophagy, pyroptosis, ferroptosis, and necroptosis [80–82].

In normal conditions, apoptosis, which helps to maintain the relative balance between cell death and regeneration, depended on the Caspase and BCL-2 family [80]. Resistance to apoptosis and maintenance of cell growth are the two main characteristics of cancer. The same HCC-derived exosomes can increase the expression of miR-21 and p-Akt in HCC cells and decrease the expression of PTEN and TETs, which inhibits cellular apoptosis [83].

Autophagy is a process of cell's self-digestion and catabolism, which degrades organelles and other subcellular structures via lysosomes and recycles the degraded products [84,85]. Previous experimental studies demonstrated that autophagy has a dual role in the progression of HCC [86]. It is shown that HCC-derived exosomal miR-3091-3p can inhibit the expression of Atg9b in liver cells, thereby inhibiting autophagosome-lysosome degradation [87].

Ferroptosis, resulting from iron-dependent lipid peroxide accumulation, is involved in tumor progression as a new type of RCD [88]. Exosomal miR-522 secreted by cancer associated fibroblasts inhibits ferroptosis of gastric cancer cells by targeting ALOX15 and blocking lipid-ROS accumulation [69]. HEK-293 T cells derived exosomes loaded with CD47 and ferroptosis inducer erastin can significantly promote ferroptosis of HCC cells [89]. However, whether exosomal miRNAs can participate in the ferroptosis of HCC is worthy of further research.

Evidence showed that pyroptosis can regulate tumor progression. In gastric cancer, ADAMTS9-AS2 serves a tumor-inhibiting role by activating NLRP3-mediated cell pyroptosis through sponging miR-223-3p [90]. But the relationship between necrosis and exosomes has not been studied sufficiently in the field of HCC. Interestingly, EV-encapsulated miR-379 derived from mesenchymal stem cells to target breast cancer cells exhibits greater areas of central necrosis [91].

The purpose of tumor treatment is to inhibit and eliminate tumor cells. And exosomes participate in these varieties of death modes by influencing related genes and molecules upstream. However, how exosomes function in these mechanisms requires further exploration. Presumably, engineered exosomes for targeted drug delivery can be used to induce cell death with high selectivity and without affecting the life cycle of normal cells.

### 3.4. Exosomal lncRNA and circRNA

Exosomes are also known to contain lncRNA and circRNA, which can function as competitive endogenous RNA. These noncoding RNAs packaged in exosomes have been recently proved to sponge-absorb miRNAs and then regulate mRNA stability and translation so as to alter HCC progression. Particularly, it contributes to HCC cells' malignant phenotypes with proliferation, migration, invasion, EMT: circ-DB [92], circ-Cdr1as [93], circ-0072088 [94], lnc85 [95], C5orf66-AS1 [96], DLX6-AS1 [97], or circ-004277 [98]. Others have been proved associated with HCC development such as LINC00511 [99], HOTAIR [100], circ-0070396 [101], CircWHSC1 [102], or circ-0074854 [103]. Besides, exosomal lncRNA and circRNA are supposed to be more sensitive and specify biomarkers than Alpha-fetoprotein (AFP). According to the differential expression of serum lnc85, AFP-negative HCC patients can be distinguished from cirrhosis and healthy control group [95]. Similarly, exosomal circ0070396 is superior to AFP in distinguishing HCC patients from healthy persons and can also be used to distinguish liver cancer patients from patients with chronic hepatitis B and patients with cirrhosis [101].

### 3.5. Exosomal Protein

Previously, it has been confirmed that exosomes from HCC cells contain thousands of proteins, which can be endocytosed by surrounding adipocytes, thereby creating a tumor microenvironment conducive to the progression of HCC [50]. Later, evidence showed that exosomal protein plays a strong regulatory role in the formation of the tumor microenvironment. Exosomal p120-catenin can inhibit the metastasis and proliferation of HCC stem cells by activating the STAT3 pathway [104]. Exosomal S100A4 is a key enhancer by upregulating OPN expression through the STAT3 signaling pathway for HCC metastasis [105]. Besides, exosomal proteins are involved in mediating the immune escape of HCC. Tumor-derived exosomal HMGB1 can expand regulatory B cells by upregulating TIM-1 and thereby promote the immune escape of HCC cells [106]. Yet there are still many gaps in the field of exosomal proteins in HCC, which need further exploration.

### 3.6. Exosomal Enzyme

Enzymes also can be delivered by exosomes to regulate HCC development. The amount and activity of the exosomal NSNase1 (neutral sphingomyelinase 1) isolated from HCC tissues are lower than normal tissues, resulting in a higher sphingomyelin/ceramide ratio and then inhibiting apoptosis and promoting the growth in HCC [107]. ENO1 can be transferred from highly metastatic HCC cells to low-metastatic-potential HCC cells through exosomes, which promote the growth and metastasis of HCC cells with low ENO1 expression by upregulating integrin *α*6*β*4 expression and activating the FAK/Src-p38MAPK pathway [108].

### 3.7. Exosomal Lipids or Other Metabolites

Metabolites such as lipids, amino acids, and inorganic substances in exosomes are involved in tumor regulations [109]. In exosomes secreted by Huh7 liver cancer cells, cardiolipin, phosphatidylserine, phosphatidylglycerol, and lanosylinositol are all enriched [110]. Ten lipid classes identified from plasma exosomes were enriched in HCC exosomes compared with non-HCC exosomes and these changes reflected alterations in glycerophospholipid metabolism, retrograde endocannabinoid signaling, and ferroptosis [111]. Although many HCC-associated lipids and other metabolites were identified from exosomes, their roles have not been elucidated and require further clarification.

## 4. Exosomes as TIME Regulators in HCC

**Figure 4:**
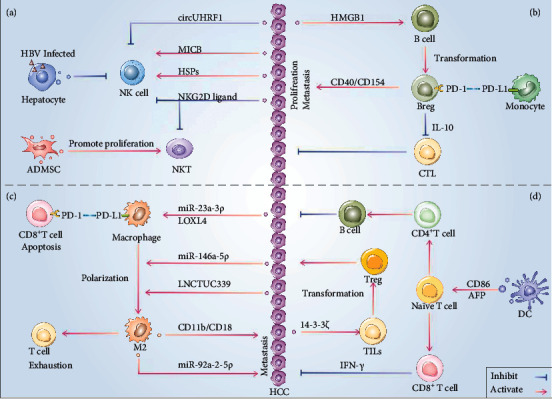
The landscape of tumor Immune microenvironment regulated by exosomes in HCC. (a) Exosomes containing specific cargos activate the function of NK cell or inhibit the function of both NK cell and NKT cell, while the function of NK cell can be inhibited by exosomes secreted by HBV infected hepatocyte and the proliferation of NKT cell can be boosted by exosomes from ADMSC. (b) Breg cells either release IL-10 to inhibit the function of CTL, which participates in reversing the progression of HCC, or express CD40/CD154 to directly stimulate the proliferation and metastasis of HCC cells, whereas PD-L1 expressed on monocytes combined with PD-1 on Breg cells lead to the exhaustion of Breg cell. (c) Macrophage expresses a higher level of PD-L1 resulting from incorporating exosomes derived from HCC, later interacting with PD-1 on CD8+T cell, and luring the apoptosis of CD8+T cell. And the same exosomes but different cargos trigger the polarization of the macrophage into the M2 subtype. (d) Exosomes containing CD86/AFP derived from DC activate naïve T cells into CD4+ T cell or CD8+T cell. Meanwhile, exosomes from HCC containing 14-3-3*ζ* transform TILs into Tregs, thus formatting the immunosuppressed environment of HCC.

Tumor cells and their surroundings form a functional unit that can be split into two types: the immune and the nonimmune microenvironment characterized by immune cells and fibroblasts separately. In the TIME, tumor cells and immune cells communicate with each other through cell contact and secretion of soluble molecules and exosomes. Therein, exosome is one of the key factors that mediate the formation of immunosuppressive microenvironment (Figure 4). In HCC, immune cell-derived exosomes play a vital role in regulating the metastasis, invasion, and immune escape [112, 113]. Therefore, in the following sections, we will elaborate the latest research progress on the mechanism of exosomes affecting various types of immune cells and explore the mutual information transmission mechanism of exosomes in the TIME of HCC.

### 4.1. Neutrophil

Neutrophils are subdivided into two subtypes according to their phenotype in the tumor microenvironment: antitumor type (N1) and protumor type (N2) [114]. The immunosuppressive TGF-*β* can promote the N2 phenotype, and the interferon *β*/*γ* (IFN*β*/*γ*) can promote the N1 phenotype [115]. As reported in HCC, the synergistic effect of TGF-*β* and Axl induces the infiltration of neutrophils into HCC tissue and promotes tumor progression [116]. HMGB1 plays a vital role in the neutrophil recruitment of acute liver injury in HCC [117]. Zhang pointed out that HMGB1 is enriched in gastric cancer-derived exosomes and can induce neutrophil autophagy and activate tumor-promoting effects [118]. But the mechanism of neutrophils phenotypes with exosomes in the shaping of the tumor microenvironment of HCC is still unclear.

### 4.2. Macrophage

The M0 macrophage can polarize to the M1 macrophage with immune surveillance effect or the M2 phenotype with a proinflammatory effect [119]. Although M1 macrophage is the cell type with a stronger capacity of phagocytosis and antigen presentation, M2 macrophage is the major phenotype in the tumor microenvironment and promotes tumor progression [120]. Multiple factors affect the polarization of macrophages, and exosomes serve as an important medium in the interaction between macrophages and HCC [121]. For example, HCC-derived exosomal TUC339 can increase the number of M2 macrophages upon treatment of IFN-*γ* and LPS, thereby regulating M1/M2 macrophages polarization [122]. The upregulated miR-146a-5p in exosomes secreted by HCC cells can drive T cell exhaustion through M2 macrophages [123].

Endoplasmic reticulum stress (ER stress), a common feature of tumor cells, can regulate tumor immunity by regulating the function of macrophages in the tumor microenvironment [124]. ER stress induced release of exosomal miR-23a-3p from HCC cells can upregulate the expression of PD-L1 in macrophages and then upregulated PD-L1 interacts with T cell PD-1 and further facilitates tumor progression by inhibiting T cell proliferation [125]. In addition, macrophages produced exosomes directly in return to regulate HCC cells. Macrophage-derived exosomal miR-92a-2-5p can increase metastasis of HCC cells by altering AR/PHLPP/p-AKT/*β*-catenin signaling pathway [126]. Polarized M2 macrophages can promote metastasis by transferring CD11b/CD18 integrins to HCC cells through exosomes [127].

Macrophages are the most numerous immune cells in the immune microenvironment. It might be a good direction in tumor treatment to enhance M1 macrophage polarization and reduce the number of M2 macrophages by using exosomes. In addition, due to the regulatory effect of exosomes in ER stress inducing the macrophages to switch into M2 macrophages, we should not ignore its important influence on tumor metastasis [128].

### 4.3. Dendritic Cell

Dendritic cell (DC) can take up and present tumor-associated antigens, while exosomes serve as transmission carriers in this process. In a subcutaneous tumor model, DCs loaded with tumor exosomes exhibited anticancer activity. The anti-HCC efficacy can be enhanced through microwave ablation technology combined with DC cell-derived exosomal vaccine [129]. DC-derived exosomes (DEX), which contain MHCI/MHCII/CD86/HSP70-90 chaperones, can trigger the activation of CD4^+^ and CD8^+^ T cells [130]. In addition, DEX containing alpha-fetoprotein (AFP) can stimulate mice with HCC to produce more IFN-*γ* and IL-2, which was conducted by the increase of CD8^+^ T cells and then decreased the regulatory T cells (Tregs), IL-10, and TGF-*β*, improving the tumor microenvironment [131].

However, the immunosuppressive effect of exosomes and DC in the local microenvironment cannot be ignored. Tumor-derived exosomes can block the differentiation of myeloid precursor cells into DC and inhibit the differentiation of bone marrow DC cells, ultimately inducing the formation of DC immune tolerance [132]. Exposure to IL-6/IL-10/PGE2/VEGF/TGF-*β* or costimulatory inhibitory pathways can induce the increased proportion of tolerogenic DCs and the apoptosis of alloreactive T cells, and the abovementioned cytokines are expressed in the progression of HCC involving exosomes (especially TGF-*β* and IL-10) [133].

In short, delving into the dual immune response of DC cells helps to reveal a more detailed DC immune landscape—that is, inducing the antitumor effect of alloreactive T cells on the one hand and mediating the immune evasion HCC by inducing the tolerance to tumor antigen on the other. Research showed that the therapeutic intervention of DC-TEXs can reduce Tregs and restore exhausted CD8^+^ T cells in mice, thereby enhancing the efficacy of sorafenib and reducing the incidence of drug resistance [134]. The immunotherapy that combines anti-PD-1 monoclonal antibodies with DEX could be supposed as a potential strategy to reshape the immune microenvironment of poorly immunogenic HCC in situ.

### 4.4. NK Cell

Natural killer cell (NK cell) as the most important cells in the natural immune system can take up tumor-derived exosomes to help tumor cells escape the immune surveillance [135]. CircUHRF1, secreted extracellularly by HCC cells, upregulates the expression of T cell immunoglobulin and mucin domain 3 (Tim-3) and inhibits the secretion of IFN-*γ* and TNF-*α* derived from NK cells, thereby inducing immunosuppression through NK cell dysfunction [34]. NKG2D ligand has strong capacities to downregulate homologous receptors and diminish the cytotoxic function of immune cells such as NK and NKT [136]. However, hepatocytes infected with HBV can release exosomes with a viral nucleic acid to induce macrophages to express NKG2D ligand and then inhibit NK cells activation, which may shape the immunosuppressive microenvironment of HCC [137].

Exosomes are a new type of secretory pathway of heat shock proteins (HSPs), and stress-inducible HSPs are considered endogenous “danger signals,” which can improve tumor immunogenicity [138]. HCC cells treated with resistant anticancer drugs such as carboplatin and irinotecan hydrochloride increase the secretion of exosomes carrying HSP60, HSP70, and HSP90. Exosomes carrying HSPs can effectively stimulate the killing effect of NK cells and the production of granzyme B [139]. Similarly, HCC-derived exosomes modified with MS-275 (an epigenetic drug) can significantly enhance the killing effect of NK cells on HCC cells by upregulating MICB and HSP70 [140].

### 4.5. T Cell

T cells can mediate the death of abnormal cells through specifically-released perforin and granzymes, or the Fas-FasL mechanism. Through a variety of mechanisms, exosomes in the TIME can affect subsets of T cells, including CD4^+^ and CD8^+^ T cells. In this part, we will introduce how T cell subsets, including CD4^+^, CD8^+^, and NKT, function in the TIME and how they are regulated by exosomes.

#### 4.5.1. CD8^+^ T Cell

We have long believed that CD8^+^ T cells can recognize and kill tumor cells. Unfortunately, most tumor-infiltrating CD8^+^ T cells lowly express cell surface molecules CD28 and CD27 and highly express immunosuppressive factors Tim-3 and PD-1 [141]. This change causes the functional exhaustion of CD8^+^ T cells and loss of antitumor effect. At the same time, exosomes derived from a variety of cells in the tumor microenvironment can regulate the loss of antitumor effects of CD8^+^ T cells. Both LOXL4 and miR-23a-3p derived from HCC cells can be delivered by exosomes and activate the expression of PD-L1 in hepatic macrophages after the exosomal internalization, thereby further inhibiting the function of CD8^+^ T cells or promoting their apoptosis [125, 142].

As NKG2D receptors are expressed on CD8^+^ T cells, NKG2D ligands and ULBP carried by exosomes can inhibit the cytotoxicity of CD8^+^ T cells through NKG2D receptors and thereby induce HCC immune escape [6, 136]. In contrast, DC cell-derived exosomes containing tumor antigens can effectively stimulate the transition of naive T cells into specific CD8^+^ T cells, maximizing the stimulation of MHCI restricted cytotoxicity against HCC [143]. It can be seen that exosomes in the tumor microenvironment have a dual regulatory effect on the function of CD8^+^ T cells.

#### 4.5.2. CD4^+^ T Cell

Although CD4^+^ T cells cannot directly kill tumor cells, CD4^+^ T cells can play an indirect role in the TIME. Evidence confirmed that HCC-derived exosomes are able to be endocytosed by CD4^+^ T cells, upregulating the inhibitory genes in CD4^+^ T cells, then leading to the loss of CD69 on their surface and decline of the function [144]. In addition, coculture of CD4^+^ T cells with HCC-derived exosomes can increase the expression level of immunosuppressive factors including IL-10, COX-2, TGF-*β*, and CTLA-4 to inhibit the immune response to HCC [145].

#### 4.5.3. Regulatory T Cell

Regulatory T cell is a special type of CD4^+^ T cell that highly expresses CD25 and specifically expresses forkhead box protein P3 (Foxp3) and can maintain immune homeostasis and immune tolerance through cell contact mechanisms or secretion of suppressive cytokines such as IL-10 and TGF-*β* [146]. Tregs inhibit the secretion of IFN-*γ*, granzyme A, granzyme B, and perforin by NK cells and CD8^+^ T cells, thereby suppressing the antitumor effects. And Tregs are involved in the formation of the immunosuppressive microenvironment in HCC through their immunosuppressive effects [147]. Interestingly, exosomes of nasopharyngeal carcinoma origin promote the proliferation of CD4^+^ CD25^−^ T cells and their conversion to CD4^+^ CD25^+^ regulatory T cells [148]. Subsequently, increased secretion of IL-10 and TGF-*β* mediates peripheral immune tolerance and immune escape of nasopharyngeal carcinoma. Similarly, Wang et al. found that HCC cells can produce 14-3-3*ζ* protein, which can be delivered to tumor-infiltrating T lymphocytes (TILs) via exosomes [149]. They then demonstrated that exosomal 14-3-3*ζ* mediates the transition of TIL from effector T cells to Tregs. The above evidence supports that exosomes transmit critical signals during the transition of T cells to Tregs.

#### 4.5.4. NKT Cell

NKT cells are a special T cell subset with characteristics of both NK cells and traditional T cells, accounting for 1/3 of liver lymphocytes. Studies have revealed that exosomes released from adipose tissue-derived mesenchymal stem cells can promote the proliferation of NKT cells and give them a stronger anti-HCC effect [150].

### 4.6. B Cell

Breg cells are a type of B lymphocytes with immunomodulatory properties, which exert immunosuppressive effects mainly by producing cytokines such as TGF-*β*, IL-10, and IL-35. Similar to Tregs, Breg cells can also promote the progression of HCC through immune suppression. Shao et al. revealed for the first time that Berg cells can directly interact with HCC cells through the CD40/CD154 signaling pathway and thereby promote the proliferation, migration, and invasion of HCC cells [151].

A special type of Breg cells expression has been reported to be functionally activated after contact with monocytes expressing PD-L1. These PD-1high Breg cells facilitate the progression of HCC by mediating the dysfunction of cytotoxic T cells through the IL-10 pathway. In addition, ordinary B cells in tumor tissues can convert into Breg cells, which depends on exosomal HMGB1 released from HCC cells [106]. The resulting Breg cells can release a large amount of IL-10 and then inhibit the function of CD8+ T, thereby mediating the HCC immune escape. As for the mechanism of how other types of B cells and Breg cells exert immunomodulation in HCC, it needs to be further studied.

## 5. Conclusion and Perspectives

Exosomes of different origins loaded with various components such as miRNA, lncRNA, circRNA, DNA, protein, or other cargos correlate with angiogenesis, chemotherapy sensitivity, and metastasis of HCC. The complex crosstalk exists among immune cells, tumor cells, tumor-associated fibroblasts, and endothelial cells in TIME, in which exosomes are the vital mediators of these interactions. In particular, exosomes, which exist in the intercellular matrix of tumor tissues, serve as a bridge for various cells in the TIME to communicate with each other. Therefore, exosomes isolated directly from tissues can more directly reflect the molecular components affecting the TIME. Single-cell sequencing is a powerful weapon to analyze the TIME and can reveal the heterogeneity and dynamic changes of tumor-infiltrating immune cells and their potential role in immunotherapy. Combining the tissue-derived exosomes with single-cell sequencing technology can help to depict a complete image of the immune microenvironment in HCC. Although progress has been made in this field, the standardized method for the extraction of HCC tissue-derived exosomes has not been established, and the separation efficiency has not been guaranteed. As a whole, this combined technology has good application prospects worthy of further exploration.

A comprehensive understanding of the TIME will facilitate the development of new therapeutic targets. Recent studies support the dual role of exosomes in TIME of HCC. On the one hand, exosomes help to promote the polarization and proliferation of Tregs and M2 macrophages, thus contributing to building an immunosuppressive microenvironment in HCC. On the other hand, exosomes can promote CTL to kill HCC cells and exert antitumor effects, as shown in Figure 4. Currently, studies supported that dendritic cell-derived exosomes can load tumor-associated antigens, play the role of antigen presentation, and then activate the immune system to recognize and kill tumors. Moreover, multiple clinical trials have also shown exciting efficacy in using them as antitumor agents [152]. In the near future, the discovery and identification of exosomes that exert immune-activating or immunosuppressive effects will be the key basis for the development of specific, targeted therapeutics drugs.

In addition, in-depth exploration of exosomes in TIME is helpful to resist drug resistance in HCC. Although there are several options of therapeutic agents, acquired drug resistance is a major obstacle to the prognosis of HCC [153]. As previously noted, exosomal miRNAs are key molecules in regulating chemotherapy resistance; therefore, the role of exosomal pathway in obtaining drug resistance phenotype of HCC cannot be ignored. Regorafenib, with a broad range of kinase inhibitory effects, has shown good efficacy in HCC, which can inhibit angiogenesis and cell proliferation and also promote antitumor immunity [154]. However, the recent study proved that exosomes produced by HCC stem cells in a RAB27A-dependent manner can be ingested by differentiated tumor cells and then upregulate the expression of their intracellular drug resistance-associated gene Nanog, thereby increasing the resistance of HCC cells to Regorafenib [155]. Next, by knocking out the RAB27 A gene, the production of exosomes of HCC stem cells could be inhibited. Furthermore, the ability of these exosomes to increase the drug resistance was significantly reduced. Clearly, blocking exosomal pathway-mediated drug resistance signaling transduction is becoming a potential therapeutic strategy against the development of drug resistance in HCC.

## Data Availability

Not applicable.

## Abbreviations

HCC: Hepatocellular carcinoma
TIME: Tumor immune microenvironment
ILV: ntraluminal vesicles
ESCRT: Endosomal sorting complexes required for transport
cfDNA: Cell-free DNA
EMT: Epithelial-to-mesenchymal transition
DEX: DC-derived exosomes
Tim-3: T cell immunoglobulin and mucin domain 3
ACD: Accidental cell death
RCD: Regulated cell death
AFP: Alpha-fetoprotein
HSP: Heat shock protein
TGF-*β*: Transforming growth factor *β*
IFN*β*/*γ*: Interferon *β*/*γ*
ER stress: Endoplasmic reticulum stress
DC: Dendritic cell
TILs: Tumor-infiltrating T lymphocytes
NK cell: Natural killer cell
Tregs: Regulatory T cells.
